# Rapid, multiplexed, whole genome and plasmid sequencing of foodborne pathogens using long-read nanopore technology

**DOI:** 10.1038/s41598-019-52424-x

**Published:** 2019-11-08

**Authors:** Tonya L. Taylor, Jeremy D. Volkening, Eric DeJesus, Mustafa Simmons, Kiril M. Dimitrov, Glenn E. Tillman, David L. Suarez, Claudio L. Afonso

**Affiliations:** 1 Exotic and Emerging Avian Viral Diseases Research Unit, Southeast Poultry Research Laboratory, US National Poultry Research Center, Agricultural Research Service, USDA, 934 College Station Road, Athens, GA USA; 2 Present Address: BASE2BIO, Oshkosh, WI USA; 3 Present Address: Office of Public Health Services, Food Safety and Inspection Services, USDA, 950 College Station Road, Athens, GA USA; 4 Present Address: Texas A&M Veterinary Medical Diagnostic Lab, Amarillo, Texas USA

**Keywords:** Next-generation sequencing, Applied microbiology

## Abstract

U.S. public health agencies have employed next-generation sequencing (NGS) as a tool to quickly identify foodborne pathogens during outbreaks. Although established short-read NGS technologies are known to provide highly accurate data, long-read sequencing is still needed to resolve highly-repetitive genomic regions and genomic arrangement, and to close the sequences of bacterial chromosomes and plasmids. Here, we report the use of long-read nanopore sequencing to simultaneously sequence the entire chromosome and plasmid of *Salmonella enterica subsp. enterica* serovar Bareilly and *Escherichia coli* O157:H7. We developed a rapid and random sequencing approach coupled with *de novo* genome assembly within a customized data analysis workflow that uses publicly-available tools. In sequencing runs as short as four hours, using the MinION instrument, we obtained full-length genomes with an average identity of 99.87% for *Salmonella* Bareilly and 99.89% for *E. coli* in comparison to the respective MiSeq references. These nanopore-only assemblies provided readily available information on serotype, virulence factors, and antimicrobial resistance genes. We also demonstrate the potential of nanopore sequencing assemblies for rapid preliminary phylogenetic inference. Nanopore sequencing provides additional advantages as very low capital investment and footprint, and shorter (10 hours library preparation and sequencing) turnaround time compared to other NGS technologies.

## Introduction

U.S. public health agencies routinely perform surveillance on microbial foodborne pathogens, and in the U.S. alone each year, approximately 1 in 6 individuals are sickened by foodborne illnesses, resulting in approximately 3,000 deaths^1^. During outbreak responses, identification of the source is instrumental to inform surveillance and public health strategies. However, specific characterization of foodborne pathogens during these surveillance programs in food production and distribution is important, as it allows for early warnings and fast removal of the contaminated food product(s) from public circulation before the development of an outbreak^1^. To that end, U.S. public health agencies have employed next-generation sequencing (NGS) using short-read sequencing technology in surveillance activities and outbreak response^2^. In addition to utilizing whole genome sequencing (WGS) for pathogen identification, more detailed information on the pathogen such as virulence, antimicrobial resistance, serotype, and inference of possible links between the sources of contamination is obtained^3^. WGS has provided faster identification of contaminated sources of outbreaks, reduced the number of illnesses and deaths due to the foodborne infections, and decreased the number of isolates needed to link the illness to the source of contamination^4,5^.

Although WGS is now a routine procedure in epidemiologic investigation and surveillance of foodborne pathogens, short-read sequencing technology faces challenges such as resolving repetitive regions, which introduce ambiguities that lead to inaccurate sequence reconstruction and incomplete and fragmented *de novo* assemblies^6–9^. These gaps can lead to the inability to determine accurate genome organization or architecture, which can be important in determining if genes are co-regulated or co-transmissible in the case of genes associated with mobile elements^10^. Even though the short-reads are accurate, closed whole genome assemblies are now commonly accomplished using a combination of both short-read (for base accuracy) and long-read sequencing technologies (for structural accuracy)^9,11,12^.

Long-read sequencing, enabled by single-molecule real-time (SMRT) sequencing technology that has been utilized since 2004, can produce reads averaging 11 kb in length, which facilitates the completion of bacterial genome assemblies that are either lacking in sequencing depth at certain repetitive areas of the genome or have areas that are missing reads completely^13^. The long-reads span across these large repetitive regions^14–16^ and can provide unbiased coverage of regions sequenced poorly with other technologies due to G/C content or other characteristics^13,17^. However, there is a need for an approach that generates inexpensive, long-read data in a short turnaround time. Such approach will offer benefits for rapid detection of an organism, complete sequencing of bacterial chromosomes and plasmids, and complementation to other sequencing technologies used in both outbreak investigations and foodborne pathogen surveillance.

The MinION (Oxford Nanopore), which is pocket-size (10 cm × 2 cm × 3.3 cm) and powered directly by a USB port from a laptop computer, is a nanopore-technology sequencer that produces long, single-molecule reads^18^ and can address these trade-offs. It is portable, field-deployable, inexpensive, and provides sequencing of both DNA and RNA in real time. Since the release of the MinION platform, bioinformatics tools have been steadily evolving, with the goal of using nanopore data to assemble accurate, whole, bacterial genomes independent of any other sequencing technology^19^. However, the relatively high error rate of the obtained raw reads is a recognized concern in nanopore sequencing data^20^.

In this study, utilizing only nanopore technology, we aimed to simultaneously sequence and assemble complete genomes of two pathogenic bacterial strains that can cause human illness worldwide, *Salmonella enterica subsp. enterica* serovar Bareilly and *Escherichia coli* O157:H7. In addition, we aimed to develop an improved bioinformatics workflow that provides accurate assemblies and to determine whether shorter sequencing time would still provide reliable results. Utilizing publicly-available tools, we report a reproducible bioinformatics workflow which assembled the circularized bacterial genomes and associated plasmids with the lowest error rate reported to date. We also demonstrate that utilizing the proposed sequencing and bioinformatics approach, sequencing of the entire chromosome and plasmid can be achieved with significantly shortened run time. This study shows that long-read nanopore sequencing can be used as a low-cost method to sequence the whole microbial genomes of foodborne pathogens. These closed assemblies provide information on genome organization and can complement existing characterization data from other technologies such as short-read sequencing.

## Materials and Methods

### Bacterial cultures and DNA extraction

The *Salmonella* Bareilly isolate (CFSAN000189) was isolated from raw shrimp in India (Biosample SAMN04364135), and the *E. coli* O157:H7 isolate (FSIS11705876) was isolated from domestic, raw, ground beef collected by the U.S. Department of Agriculture Food Safety and Inspection Services (USDA-FSIS) as part of routine sampling of a U.S. establishment (Biosample SAMN08167607). Both bacterial isolates were grown on sheep blood agar (SBA) for 24 hours at 35 °C. Total DNA from each isolate was extracted using the DNeasy Blood and Tissue Kit (Qiagen, USA) following manufacturer’s instructions. DNA concentrations throughout the experiment were determined by using the Qubit® dsDNA HS Assay Kit on a Qubit® fluorometer 3.0 (Thermo Fisher Scientific, USA).

### Library preparation and MinION sequencing

The 1D gDNA long read selection protocol was used with the SQK-LSK108 kit (Oxford Nanopore Technologies, UK) to prepare MinION-compatible libraries. The DNA shearing step was eliminated from the protocol with the aim of selecting for very long reads. Approximately, 2 µg of *E. coli* DNA and 2 µg of *Salmonella* DNA in a total of 100 µL each were added to the NEBNext® Ultra™ II End Repair/dA-Tailing module (New England Biolabs, USA) for end repair and dA-Tailing, following manufacturer’s instructions, and purified using Agencourt AMPure XP beads (Beckman Coulter, USA). Each purified, end-prepped DNA product was barcoded using a separate barcode from the 1D Native barcoding kit (EXP-NBD103, ONT) and following the 1D Native barcoding genomic DNA protocol. The samples were then bead-purified (Beckman Coulter), and equimolar amounts of each barcoded sample were pooled together for a final quantity of 700 ng. Adapters were ligated to the pooled sample using Blunt/TA ligase (New England Biolabs) following the 1D gDNA long read selection protocol. The MinION device was used to sequence the created library on a new FLO-MIN106 R9.4 flow cell^21,22^. The standard 48 hr 1D sequencing protocol was initiated using the MinKNOW software (ONT, UK). Average quality and coverage of the raw sequencing data were determined using CG-pipeline^23^.

### MiSeq sequencing and quality control

To verify the newly developed approach used in this study, libraries for short-read WGS of the *Salmonella* Bareilly and *E. coli* isolates were prepared using the Nextera XT kit (Illumina, USA) according to the manufacturer’s protocol. The libraries were loaded separately into a single flow cell of the 300 and 500 cycle MiSeq Reagent Kits v2 for *Salmonella* Bareilly and *E. coli*, respectively, and paired-end sequencing (2 × 150 bp for *Salmonella* Bareilly and 2 × 250 bp for *E. coli*) was performed on the MiSeq instrument (Illumina, USA). The produced raw data were analyzed using SPAdes version 3.71^24^. Average quality and coverage of the raw sequencing data were determined using CG-pipeline^23^.

### MinION bacterial bioinformatics workflow for whole genome assembly

To analyze the MinION sequencing data, a customized workflow was developed. For subsequent time analysis, the data was also analyzed at intervals from the start of the sequencing – at 15, 30, 60, 120, 240, 480, 960 and 1500 minutes (mins). Reads were basecalled using Albacore (v 2.0.2b, Oxford Nanopore Technologies) and subsampled for assembly using Filtlong (v.0.2.0)^25^ to a target depth of 75X with read quality weighted more heavily than length (‘mean_q_weight 5’). The filtered reads were assembled using the Unicycler pipeline (v.0.4.7)^26^. This pipeline utilizes a minimap/miniasm/racon iterative approach to assemble long-read-only data. Since Unicycler sometimes fails to detect valid end overlaps, assemblies were circularized using a custom script based on minimus2^27^ (available in the workflow source repository). Circular contigs were rotated to start at a fixed position based on the reference. The consensus sequences were subjected to two rounds of polishing using Nanopolish (v.0.10.2)^28^, for which the full run (subject to time-based sub-setting but prior to Filtlong subsampling) was used, and Benchmarking Universal Single-Copy Orthologs (BUSCO v.3.0.2)^29^ was used to evaluate the completeness of coding sequences and degree of gene fragmentation in the polished assemblies. To evaluate assembly accuracy, two procedures were used for the *Salmonella* Bareilly isolate, which has previously been sequenced and published^30^. DNAdiff (MUMMER v.3.23)^31^ was used to evaluate both base-level and structural accuracy in the MinION assembly compared to the published reference. For the *E. coli* isolate, lacking a published reference, Illumina MiSeq reads were mapped to the assembly using BWA (v0.7.17), and LoFreq (v.2.1.3.1)^32^ was used to call single nucleotide polymorphisms (SNPs) and small indels, from which the assembly accuracy was calculated. Utilizing the short-read data, Pilon (v1.2.2)^33^ was used to error-correct small errors (‘--fix bases’) in the assemblies using existing short-read data from the same isolates (SRA accession SRR498276 for *Salmonella* Bareilly; SRA accession SRR6373397 for *E. coli* O157:H7).

### MinION annotation

The polished-MinION assemblies after 4 hours of sequencing were initially annotated using the “Annotate From” tool within Geneious 11.1.5 and the published *Salmonella* Bareilly strain CFSAN000189 (GenBank Accession NC_021844) and *E. coli* O157:H7 strain 9234 (GenBank Accession CP017446) sequences as references. ResFinder v.3.1 was used to locate any antimicrobial resistance genes and any point mutations that would result in antimicrobial resistance^34^. Additionally, to confirm the 4-hour assembly annotation, the pilon-corrected, final genome sequences were submitted to GenBank to be processed through the NCBI Prokaryotic Genomic Annotation Pipeline (PGAP) before being released.

### Phylogenetic analysis

Twenty-three *Salmonella* reference datasets (Supplementary Table S1) used in tracing a foodborne outbreak in the U.S that were previously published^30,35^ were downloaded. For the MinION-only data to be comparable, the eight sub-sampled (15 mins to 1500 mins) unpolished S. Bareilly assemblies obtained in this experiment were used to generate simulated Illumina datasets using ART (150 × 2, 50X coverage, MiSeq platform, 300 bp mean fragment length, 50 bp standard deviation)^36^. All datasets were analyzed with a SNP-calling pipeline using strain CFSAN000212 as a reference. Briefly, reads were optionally trimmed using Trim Galore (Illumina datasets), aligned to the reference using BWA-MEM^37^, SNPs were called using LoFreq^32^, and filtered using local scripts according to specific criteria. For Illumina datasets, the VCF files were filtered by removing indels as well as any SNPs with an alternate allele frequency of <90%. Sites meeting one or more of the following criteria were flagged as suspect, and these loci were ignored during matrix generation: (i) sites within 3 bp of a homopolymeric stretch of 4 bp or more; (ii) sites occurring in a variant cluster (multiple variants within 2 bp of each other; (iii) sites within 10 bp of a dam or dcm methylation motif; and (iv) sites with observed A- > G or T- > C transition mutations. The remaining SNPs were used to create a matrix of variable sites for phylogenetic reconstruction. MEGA6 (v.6.06) was used to generate a Neighbor-joining SNP trees utilizing the Maximum Composite Likelihood model with 1000 bootstrap iterations^38^. Three separate trees were constructed. The first tree was built using the SNP matrix obtained from the 23 *Salmonella* reference datasets^35^ (Supplemental Table S1). The second tree was constructed by replacing the reference Illumina data of the CFSAN000189 strain with the MinION-only data obtained by sequencing the same strain in this study (240 and 1500 mins time points were used). A third tree that contained both the Illumina and the MinION-only data of the CFSAN000189 strain was also built for comparison.

### Availability of workflows, tools and code

The full NextFlow workflow, Conda environment configuration, and other associated code used in the analyses are publicly-available on GitHub (https://github.com/jvolkening/minion_bacterial).

## Results

### Analysis of MinION and MiSeq raw data

Before subsampling of the reads, the raw MinION sequencing data was used to estimate the mean depth for *Salmonella* Bareilly and *E. coli*, respectively. A total of 2.8 billion bases from 333,298 *Salmonella* Bareilly reads, with an average read length of 8638 nucleotides (nt), yielded a mean depth of 599X. For *E. coli*, a total of 3.8 billion bases from 429,909 reads with an average read length of 8979 nt were sequenced, and the mean depth was calculated to be 692X (Table 1). The shortest MinION read was 85 nt, which was from the *E. coli* isolate, while the longest read was from *Salmonella* Bareilly and was 129,119 nt. Both sets of MinION data had a mean read quality score above the standard (Q ≥ 10).

**Table 1:** Comparison of the final raw data from MinION and Illumina.

Sequence Method	Average Read Length	Total Bases	Min Read Length	Max Read Length	Average Read Quality	Read Number	Mean Depth
MiSeq (*Salmonella*)	149.51	288,633,579	35	151	36.66	1,930,511	57.72
MinION (*Salmonella*)	8638.36	2,879,148,408	113	120,119	19.36	333,298	599.06
MiSeq (*E. coli*)	242.61	556,035,081	35	251	34.96	2,291,825	111.2
MinION (*E. coli*)	8979.55	3,860,389,678	85	112,643	19.38	429,909	692.19

^a^MiSeq Quality Standards = Q ≥ 30.

^b^MinION Quality Standards = Q ≥ 10.

Illumina MiSeq data was also analyzed using the same bioinformatics tool. The MiSeq raw data had a depth of 57X for *Salmonella* Bareilly and 111X for *E. coli*. This sequencing technology produced 288 million bases from 1,930,511 *Salmonella* Bareilly reads, with an average read length of 150 nt. For *E. coli*, a total of 556 million bases from 2,291,825 reads were sequenced that had an average read length of 243 nt (Table 1). The minimum read length from both sets of bacterial sequences was 35 nt, while the longest was 151 nt for *Salmonella* Bareilly and 251 nt for *E. coli*; the MiSeq mean read quality was above the Q30 benchmark.

### Assembly of MinION sequencing data

The raw MinION data for both isolates were subsampled on the basis of cumulative run time in order to simulate the effect of run time on final assembly quality. Subsets of reads generated in the first 15, 30, 60, 120, 240, 480, and 960 mins, in addition to the full run length of 1500 mins, were analyzed (Table 2). Four hours (240 mins) was determined as the shortest run time sufficient to assemble circular sequences from all chromosomes and plasmids from both isolates and represented a point after which longer run times resulted in significantly diminishing gains in final accuracy (Supplemental Fig. S1). Detailed data at each of the other run time subsets is available in Tables 2–4; however, the following analyses herein refer to the data collected in the first four hours of sequencing.

**Table 2:** Assembly data for MinION sequencing.

Duration (min)	Reads	Subsampled Reads	Assembly Size	Circular Contigs^a^	Linear Contigs	Longest Contig	Longest Circular Contig	NG50^b^	Average identity in %	Reference Coverage
***Salmonella enterica subsp. enterica*** **serovar Bareilly**										
15	7229	7229	1135723	0	19	163786	0	0	99.13	24.36
30	14888	14888	4577215	0	18	841969	0	471499	99.55	95.38
60	29132	29132	4722179	1	0	4722179	4722179	4722179	99.79	98.4
120	51226	51226	4805334	2	0	4723663	4723663	4723663	99.84	100
**240**	**84156**	**28492**	**4806150**	**2**	**0**	**4724389**	**4724389**	**4724389**	**99.87**	**100**
480	132137	20193	4806518	2	0	4724724	4724724	4724724	99.87	100
960	248910	16221	4806892	2	0	4725103	4725103	4725103	99.89	100
1500	333298	15249	4806995	2	0	4725191	4725191	4725191	99.89	100
***Escherichia coli*** **O157:H7**										
15	8731	8731	1352560	0	19	154626	0	0	99.18	
30	18053	18053	5141583	0	14	1565772	0	518218	99.63	
60	35335	35335	5481126	1	0	5481126	5481126	5481126	99.82	
120	62415	60362	5570410	1	1	5481662	5481662	5481662	99.87	
**240**	**103681**	**19589**	**5577045**	**2**	**0**	**5482542**	**5482542**	**5482542**	**99.89**	
480	164641	15265	5577346	2	0	5482831	5482831	5482831	99.90	
960	317698	12941	5577818	2	0	5483284	5483284	5483284	99.91	
1500	429909	12403	5577934	2	0	5483397	5483397	5483397	99.91	

^a^Two circular contigs indicates both the chromosome and the plasmid.

^b^NG50 - 50% of the entire assembly is contained in contigs or scaffolds equal to or larger than this value.

**Table 3:** *Salmonella* Bareilly MinION sequencing data analyzed for completeness and accuracy before and after two rounds of polishing.

Seq Duration (min)	Reference Coverage	Avg. ID	rel^a^	t^b^	inv^c^	ins^d^	ins sum	SNPs/kb^e^	Indels/kb^f^	BUSCO complete^g,h^	BUSCO fragmented^g,i^	BUSCO missing^g,j^
**No Polishing**												
15	24.36	98.6	0	0	1	12	506	4.04	9.43	0.02	0.07	0.91
30	95.38	99.05	0	0	1	10	292	3.01	6.43	0.14	0.51	0.35
60	98.4	99.3	0	0	1	0	0	2.54	4.46	0.19	0.59	0.22
120	100	99.32	0	0	1	1	3613	2.43	4.39	0.2	0.57	0.22
**240**	**100**	**99.37**	**0**	**0**	**1**	**1**	**3612**	**2.41**	**3.91**	**0.21**	**0.57**	**0.22**
480	100	99.37	0	0	1	1	3618	2.42	3.89	0.21	0.55	0.24
960	100	99.36	0	0	1	1	3612	2.38	4.05	0.2	0.57	0.23
1500	100	99.37	0	0	1	2	3606	2.4	3.85	0.23	0.55	0.22
**One Round of Polishing**												
15	24.36	99.1	0	0	1	11	494	2.19	6.52	0.04	0.11	0.85
30	95.38	99.52	0	0	1	10	292	1.22	3.48	0.32	0.5	0.18
60	98.4	99.77	0	0	1	0	0	0.6	1.72	0.46	0.44	0.1
120	100	99.81	0	0	1	1	3610	0.49	1.38	0.54	0.38	0.08
**240**	**100**	**99.84**	**0**	**0**	**1**	**1**	**3616**	**0.42**	**1.14**	**0.61**	**0.32**	**0.07**
480	100	99.86	0	0	1	1	3616	0.4	1.08	0.61	0.33	0.06
960	100	99.85	0	0	1	1	3612	0.44	1.02	0.62	0.33	0.06
1500	100	99.86	0	0	1	2	3610	0.41	1.01	0.62	0.31	0.06
**Two Rounds of Polishing**												
15	24.36	99.13	0	0	1	11	492	2.06	6.35	0.05	0.12	0.84
30	95.38	99.55	0	0	1	10	292	1.1	3.33	0.34	0.48	0.18
60	98.4	99.79	0	0	1	0	0	0.48	1.61	0.5	0.42	0.08
120	100	99.84	0	0	1	1	3610	0.34	1.26	0.58	0.35	0.07
**240**	**100**	**99.87**	**0**	**0**	**1**	**1**	**3616**	**0.26**	**1.03**	**0.65**	**0.3**	**0.05**
480	100	99.87	0	0	1	1	3616	0.24	0.99	0.66	0.29	0.05
960	100	99.89	0	0	1	1	3612	0.23	0.89	0.67	0.28	0.04
1500	100	99.89	0	0	1	2	3610	0.23	0.86	0.69	0.27	0.04

^a^Relocations – rearrangement of genetic material within a chromosome or between chromosomes.

^b^Translocations- rearrangement of parts between nonhomologous chromosomes.

^c^Inversions - rearrangement in which a segment of a chromosome is reversed end to end.

^d^Insertions - the addition of a larger nucleotide sequence into a chromosome.

^e^SNPs/kb – single nucleotide polymorphisms per kilobase.

^f^Indels/kb – insertions or deletions per kilobase.

^g^BUSCO- Benchmarking Universal Single-Copy Orthologs.

^h^Complete-fraction of expected gene complement with full-length reading frames.

^i^Fragmented- decreased length alignment of genes.

^j^Missing- no significant matches.

**Table 4:** *E. coli* MinION sequencing data analyzed for completeness and accuracy before and after two rounds of polishing.

Seq Duration (min)	Avg ID	SNPs/kb^a^	indels/kb^b^	BUSCO complete^c,d^	BUSCO fragmented^c,e^	BUSCO missing^c,f^
**No polishing**						
15	98.62	4.06	9.69	0.01	0.06	0.93
30	99.16	2.67	5.71	0.13	0.5	0.37
60	99.36	2.31	4.07	0.2	0.57	0.23
120	99.4	2.22	3.74	0.23	0.55	0.22
**240**	**99.39**	**2.22**	**3.86**	**0.23**	**0.54**	**0.23**
480	99.38	2.21	4	0.22	0.57	0.21
960	99.41	2.25	3.71	0.23	0.55	0.22
1500	99.4	2.24	3.72	0.22	0.58	0.2
**One round of Nanopolish**						
15	99.13	2.11	6.61	0.04	0.11	0.86
30	99.6	1.02	2.96	0.35	0.46	0.19
60	99.79	0.55	1.51	0.51	0.41	0.08
120	99.85	0.39	1.14	0.58	0.35	0.07
**240**	**99.86**	**0.37**	**1**	**0.64**	**0.31**	**0.04**
480	99.87	0.35	0.94	0.66	0.3	0.04
960	99.88	0.35	0.87	0.66	0.3	0.04
1500	99.88	0.35	0.87	0.64	0.31	0.05
**Two rounds of Nanopolish**						
15	99.18	1.92	6.31	0.04	0.11	0.86
30	99.63	0.88	2.81	0.37	0.44	0.19
60	99.82	0.41	1.39	0.57	0.36	0.06
120	99.87	0.26	1.04	0.64	0.3	0.06
**240**	**99.89**	**0.2**	**0.89**	**0.69**	**0.28**	**0.04**
480	99.9	0.19	0.8	0.72	0.26	0.02
960	99.91	0.19	0.75	0.73	0.25	0.02
1500	99.91	0.18	0.74	0.73	0.24	0.03

^a^SNPs/kb – single nucleotide polymorphisms per kilobase.

^b^Indels/kb – insertions or deletions per kilobase.

^c^BUSCO- Benchmarking Universal Single-Copy Orthologs.

^d^Complete-fraction of expected gene complement with full-length reading frames.

^e^Fragmented- decreased length alignment of genes.

^f^Missing- no significant matches.

The MinION sequencing data was assembled using a custom Nextflow^39^ workflow that utilized publicly-available tools. Filtlong quality- and length-based subsampling resulted in 28,492 reads for the *Salmonella* Bareilly isolate, which were assembled into two circular contigs, the chromosome (4,724,389 bp) and plasmid (81,761 bp), with an average nucleotide identity of 99.87% and coverage of 100% compared to the reference genome (Table 2). For the *E. coli* isolate, 19,589 subsampled reads produced two circular contigs, the chromosome (5,482,542 bp) and plasmid (94,503 bp), with an average nucleotide identity of 99.89% compared to the available MiSeq data of the same bacterium (Table 2).

**Figure 1:**
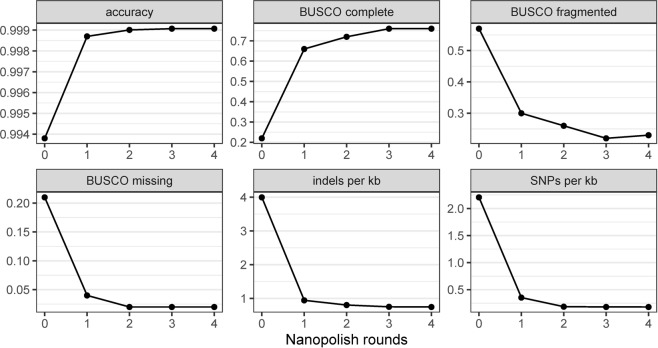
Polishing Results of the MinION-only Assemblies Using Multiple Rounds of Nanopolish. Due to the errors remaining in the MinION-only assemblies, a signal-level consensus software, Nanopolish, was used to increase the assembly accuracy. The overall accuracy, the Benchmarking Universal Single-Copy Orthologs (BUSCO) completeness, BUSCO Fragmented, BUSCO Missing, number of indels per kb, and number of SNPs per kb are shown after 0, 1, 2, 3 and 4 rounds of Nanopolish. After two rounds of polishing, the overall accuracy and the number of Indels and SNPs per kb did not considerably change.

The final genome assemblies utilized two rounds of polishing using Nanopolish, which represented, by far, the most time-consuming and resource-intensive portion of the analysis workflow. However, it also increased the overall accuracy (Fig. 1a) due to a decrease in both SNPs (Fig. 1b) and chromosomal insertions or deletions (Fig. 1c). The largest gains in accuracy were achieved from the first round of polishing, while much less but still noticeable improvement was achieved with the second round, particularly when examining completeness of genome annotation as measured by BUSCO. However, further rounds (>2) of polishing did not significantly impact the overall assembly (Fig. 1). The central processing units (CPU) time and memory consumption for the assembling and polishing steps of the workflow can be found in Supplemental Table S2.

Detailed statistics of the assemblies’ accuracy are provided in Tables 3 and 4. For the *Salmonella* Bareilly assembly after 4 hours of sequencing, the rate of single nucleotide polymorphisms (SNPs) per kilobase (kb) decreased from 2.41 to 0.42 after one round of polishing and to 0.26 after two rounds of polishing. At the same time point, the insertions or deletions (indels) per kb decreased from 3.91 to 1.14 and 1.03 after one and two rounds of polishing, respectively (Table 3). For the *E. coli* assembly at the same time point, the SNPs per kb decreased from 2.20 to 0.37 after only one round of polishing and to 0.2 after two rounds of polishing. The indels per kb also decreased from 3.86 to 1 to 0.89 (Table 4). Additionally, the BUSCO tool was used to further analyze the polished data to determine the completeness of the gene content based on quality and length of alignment. The “BUSCO completeness” (fraction of expected gene complement with full-length reading frames) value of both bacterial assemblies and the rounds of polishing were directly related, increasing from 21 and 23% for the Salmonella and *E. coli* assemblies, respectively, with no polishing to 65 and 69% after two rounds of polishing; the BUSCO fragmented (decreased length alignment of genes) and BUSCO missing (no significant matches) values decreased correspondingly (Tables 3 and 4).

### MinION assembly annotation

Both 4-hour MinION assemblies, after two rounds of polishing with Nanopolish, were annotated using Geneious and the most closely related, published, annotated genomes for each bacterial species. Since the *Salmonella* Bareilly genome was already completed and closed by a hybrid Illumina/PacBio approach and published, we confirmed that the Geneious genome annotation of the sequence of the same bacterium produced by MinION was accurately reconstructed (loci of protein-coding genes), by using the PGAP annotations tool on the final, corrected assembly; for example, but not limited to, the two major serotyping antigens located on the chromosome: the flagellin FliC CDS and the O-antigen polymerase.

**Figure 2:**
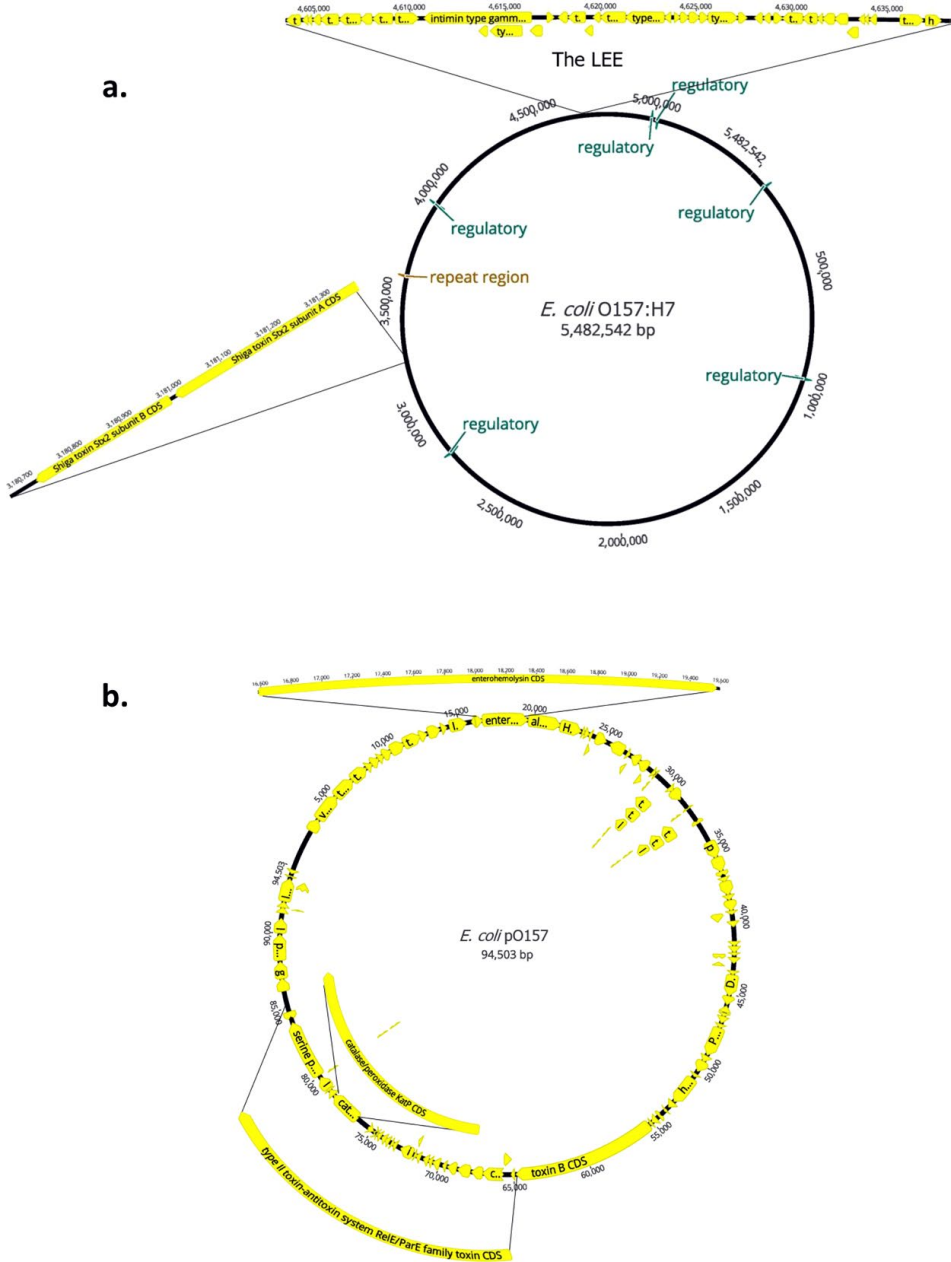
Annotation of the MinION assembly of *Escherichia coli*. (**a**) The *E. coli* O157:H7 chromosome was sequenced and assembled into a final consensus of 5,482,542 nucleotides. The annotation of the genome provided the location of 5,748 coding sequences (CDS), 106 tRNAs, 29 rRNAs, 6 regulatory regions, and 1 repeat regions. For imaging purposes, only the 6 regulatory regions (green), the one repeat region (brown) and the CDS of two virulence factors (yellow) are shown magnified. The LEE (locus of enterocyte effacement) is highlighted at position 4,603,699 to 4,636,299, and the Shiga Toxin subunits are shown at position 3,181,004 to 3,180,992 for demonstration purposes. (**b**) The *E. coli* pO157 plasmid was sequenced and assembled into a final consensus of 94,503 nucleotides. The annotation shows all 124 coding sequences (CDS) in yellow. The CDS of three well-known virulence factors are highlighted: hemolysin (*ehx*) at position 16,584 to 19,578, catalase-peroxidase (*katP*) at position 76,704 to 78,356, and the type II secretion system (T2SS) at position 64,056 to 85,694 for demonstration purposes.

The presence of major virulence factors in the *E. coli* MinION-only assembly were identified, as well as genes that would cause possible antimicrobial resistance, using Geneious (Fig. 2a,b). The locus of enterocyte effacement (LEE), one of the major virulence factors of enterohemorrhagic *E. coli*^40,41^ that includes the gene intimin for adhesion and the type III secretion system, was annotated between positions 4,603,699 and 4,636,299 in this MinION-only assembly (Fig. 2a). Additionally, the genes expressing the Shiga toxins (Stx), responsible for causing host cell damage^40,42^, were annotated from position 3,181,004 to 3,181,963 for Stx subunit A and from position 3,180,723 to 3,180,992 for Stx2 subunit B (Fig. 2a). The multidrug resistance gene Mdf(A), which encodes a membrane protein that confers resistance to a multitude of clinically important drugs, including macrolides, lincosamides, and streptogramin B^43^, was also identified at position 1,012,477 to 1,013,709. No other genes or point mutations that would confer antimicrobial resistance were detected. Not only was the full-length chromosome of this *E.coli* O157:H7 isolate sequenced using MinION, but also the full-length pO157 (Fig. 2b). Genes that encode *E. coli* O157-specific virulence factors^40^, such as hemolysin (*ehx*), catalase-peroxidase (*katP*), and the type II secretion system (T2SS) were identified in the sequenced plasmid.

### Additional polishing of the MinION assemblies with MiSeq Data

One of the main objectives of the presented work is to determine if MinION alone can be utilized to obtain fully closed genomes and plasmids from important foodborne pathogens. However, for submission of final sequences to GenBank, the most accurate assemblies attainable were used. To this end, for both samples, assemblies produced using the full run length, were utilized and further error-corrected using Pilon, together with available MiSeq data. Pilon utilizes the low error rate of Illumina reads mapped to the draft assembly to drastically improve the local accuracy of the final sequence. The error rate for both samples after Pilon polishing decreased, with accuracy rates of 99.99% and 100%, and BUSCO completeness rates of 99.7% and 99.99% for *Salmonella* and *E.coli*, respectively. There were also a reduction in SNPs per kb to 0.002 and 0.001 and indels per kb to 0.008 and 0.002 for *Salmonella* and *E. coli*, respectively. The assembled, polished, and short-read error-corrected data from the full 25-hour run were the final assemblies annotated and submitted to GenBank (Accession numbers CP034177- CP034178 and CP035545-CP035546, Bioproject PRJNA498670).

### Phylogenetic inference (SNP tree)

**Figure 3:**
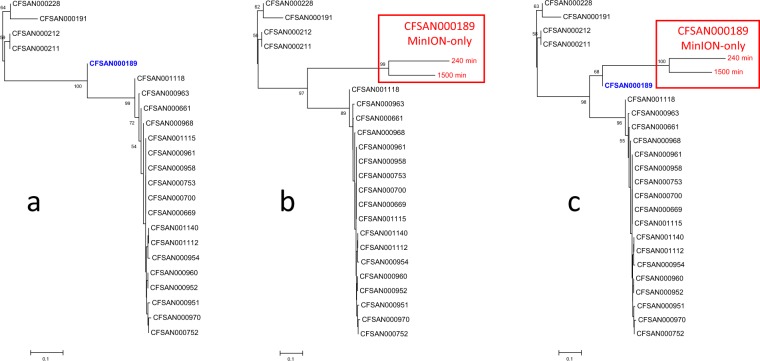
SNPs trees of Salmonella reference datasets and data obtained with MinION. (**a**) Constructed with SNPs of twenty-three *Salmonella* reference datasets which were used for phylogenetic pipeline validation for foodborne pathogen surveillance^35^; (**b**) The CFSAN000189 data is replaced with SNPs from the 240 mins and 1500 mins MinION-only assemblies obtained in this study; (**c**) The tree includes both the reference dataset and the MinION-only data for the CFSAN000189 strain along with the SNPs of the remaining 22 *Salmonella* reference datasets.

The constructed SNPs trees are presented in Fig. 3. The tree built with the reference *Salmonella* datasets used for phylogenetic pipeline validation for foodborne pathogen surveillance^35^ is depicted in Fig. 3a. To demonstrate the potential of the MinION-only sequencing for rapid preliminary phylogenetic inference, the SNPs data for strain CFSAN000189 sequenced in this study, was replaced with the data from our assemblies, and the resulting tree is depicted in Fig. 3b. For simplicity, 240 mins and 1500 mins timepoints were used for the reconstruction. The comparison between the trees built with the reference datasets and the tree utilizing the MinION-only data for the CSAFN000189 strain demonstrates topological congruence between the trees. The results using all eight time points showed identical topology (data not shown). An additional tree using both the Illumina and the MinION data of strain CFSAN000189 was constructed (Fig. 3c). The results showed clustering in a monophyletic branch (98% branch support) of all CFSAN000189 data. The constructed trees were also congruent to the standard tree provided by Timme *et al*.^35^.

## Discussion

In this study, we demonstrate that long-read, nanopore sequencing technology can be used as a single tool to sequence full length bacterial chromosomes and plasmids. Utilizing a customized workflow, optimized and tailored for bacterial sequencing results, and MinION-only data, whole genome sequences with as little as 0.1% error rate, were produced. These assemblies are 0.4% and 3.1% more accurate compared to previous reports^10,19^. The tools used in our customized bioinformatics workflow are publicly-available^25,26^ and the Conda environment configuration, along with other associated code used in the analyses, are also provided for public use.

Using MinION sequencing alone, two completely closed contigs, one chromosome and one plasmid for each pathogen, were assembled. This capability and the low cost make the MinION highly accessible as both a primary sequencing platform, as well as a secondary platform to complement laboratories’ existing sequencing infrastructure. The initial investment required for the MinION is drastically lower (starter pack costs $1000) than other sequencing technologies, each flow cell can be used for multiple runs, and samples can be multiplexed together per run to further reduce the cost^21,44^. Based on the results of barcoding and simultaneous sequencing of two whole bacterial genomes and plasmids shown here, we estimate that six bacterial samples could be multiplexed together to further decrease cost and sequenced in approximately 16 hours to obtain complete genomic data with high accuracy.

The effects of increased sequencing run lengths, different criteria and weights to subsample data for assembly, and increased rounds of polishing, were examined for their effect on the final assembly completeness and accuracy. Filtlong subsampling is not random but keeps the longest and highest quality reads from the input, which targets maximum sequencing depth (total bases). It was observed that the nanopore reads were long enough on average that over-aggressive length-based filtering resulted in reduced representation. Such extensive subsampling would result in less complete assembly of small plasmids, which can contain virulence factors of great interest for diagnostic and food safety purposes. It therefore proved critical to evaluate filtering and subsampling criteria to take full advantage of the technology. Read quality was weighted more heavily than length, as testing showed this was necessary to retain sufficient coverage of small plasmids.

Our results suggest that at least one round of polishing with Nanopolish is needed to achieve acceptable accuracy, and a second round provides additional improvement if the near-doubling of the analysis time is warranted. The data in Supplemental Table S2 are provided when only one core is utilized, but due to the wide availability of high-performance computers, the analysis time for two rounds of polishing can decrease to 6 hours using 124 cores, for example. In MinION-only assemblies, it is known that putative pseudogenes caused by systematic indel errors (often near homopolymeric tracts^19,45^), leading to reading frame shifts can be an issue, as evident from the “BUSCO fragmented” column in Tables 3 and 4. Even after polishing, this value was observed to be greater than 20% of expected coding genes, which must be taken into consideration during annotation. However, the polished assemblies, with only 0.1% error are accurately reconstructed and reveal serotype and important genes responsible for the virulence, metabolism, defense, and pathogenesis of the bacterium.

In outbreak situations, a rapid turn-around time is necessary. Therefore, polymerase chain reaction (PCR), real-time PCR assays, and other rapid diagnostic assays are still deployed. However, WGS has become routine in use and coupled with proper bioinformatics analysis can provide complete genome sequences in a couple of days^2^. With the MinION platform and sufficient computational resources (which can be cloud-based and thus widely available), basecalled sequence data can be analyzed in near-real-time as it comes off of the machine^46^. Therefore, the MinION can be used for rapid diagnostics as initial sequencing data from pure cultures can be provided in approximately 9 to 10 hours^47^. Furthermore, the MinION-only results have potential for rapid preliminary phylogenetic inference as demonstrated by the congruent topology between trees (and to the standard tree provided by Timme *et al*.^35^) built with the Illumina and the MinION-only data (only after four hours of sequencing). Of note, due to the higher MinION sequencing error rate, the distances between the MinION-only results and references were higher compared to the reference tree. However, the nanopore and bioinformatics are constantly improving, the quality and accuracy of the sequences steadily increase, and the MinION-only results would likely be epidemiologically informative in the near future. The complete MinION data can be further analyzed and polished after the entire sequencing run to obtain more accurate whole genomes that provide detailed data on subtyping, virulence genes, antimicrobial resistance genes, and other genetic characteristics. Same-day detection of antimicrobial resistance genes with 99.75% accuracy (with polishing) after enriching for plasmid DNA and MinION sequencing has been recently demonstrated^48^.

In conclusion, this low-cost, rapid, random-priming nanopore sequencing approach, coupled with our customized workflow, provides sufficient data where complete genomes, including plasmids, can be assembled into a single contiguous sequence with 99.89% accuracy (highest reported-to-date). These data allowed accurate gene identification and genomic organization without the need for additional sequencing tools to close gaps that are required by other sequencing methods. As the nanopore chemistry and bioinformatics continue to evolve, this method is promising in providing a sufficient amount of accurate data to complement the current sequencing methods by resolving repetitive regions of the genome, which will be instrumental in increasing the number of available complete genome assemblies.

## Supplementary information

Supplementary

## Data Availability

The final assemblies generated during the current study are available in GenBank (Accession CP034177- CP034178 and CP035545-CP035546). The raw data generated during the current study are available under BioProject number PRJNA498670, BioSamples numbers SAMN04364135 and SAMN08167607, and SRA Accession numbers SRR9603470 and SRR9603471.
