# Supplementary material for: Rapid, multiplexed, whole genome and plasmid sequencing of foodborne pathogens using long-read nanopore technology
Source: Sci Rep. 2019 Nov 8;9:16350. doi: 10.1038/s41598-019-52424-x (PMC6841976; doi:10.1038/s41598-019-52424-x)
Supplement: Supplementary file 1 — Supplementary [file 41598_2019_52424_MOESM1_ESM.pdf]

Supplementary Files

Rapid, multiplexed, whole genome and plasmid sequencing of foodborne pathogens using long-read nanopore technology

Tonya L. Taylor[1], Jeremy D. Volkening[2], Eric DeJesus[3], Mustafa Simmons[3], Kiril M. Dimitrov[1], Glenn E. Tillman[3], David L. Suarez[1] and Claudio L. Afonso[1*]

1. Exotic and Emerging Avian Viral Diseases Research Unit, Southeast Poultry Research Laboratory, US National Poultry Research Center, Agricultural Research Service, USDA, 934 College Station Road, Athens, GA, USA.
2. BASE$_2$BIO, Oshkosh, WI, USA.
3. Office of Public Health Services, Food Safety and Inspection Services, USDA, 950 College Station Road, Athens, GA, USA

*Corresponding author: **Claudio L. Afonso**. Phone: +1 (706) 546 – 3642. Email: Claudio.Afonso@ars.usda.gov. Mailing address: USDA ARS, Southeast Poultry Research Laboratory, 934 College Station Rd., Athens, GA 30605. Fax: (706) 546-3161.

Supplemental Figure S1. MinION sequencing run time versus accuracy of polished (2x) assemblies.

The raw MinION sequencing data for both isolates were subsampled based on cumulative run time in order to simulate the effect of run length on polished assembly quality. At the 240-minute time point, two circularized contigs, one chromosome and one plasmid, from both isolates were fully sequenced, and the accuracy, after two rounds of polishing, levels off and does not significantly increase with longer run times.

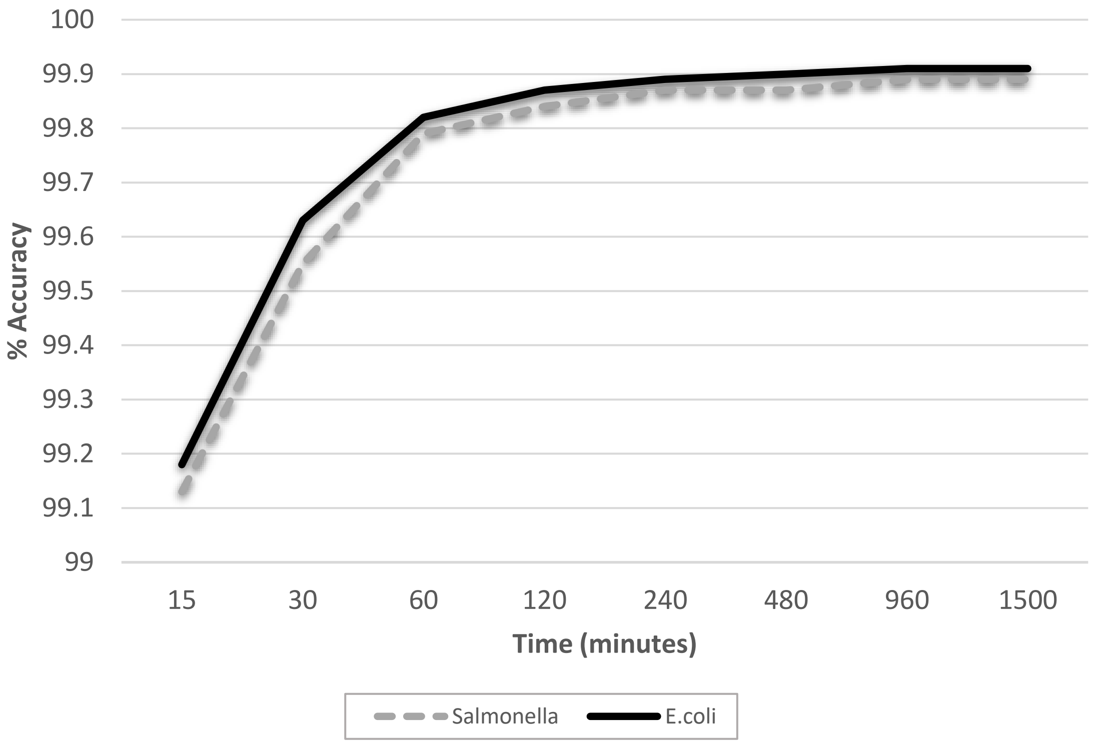

Supplemental Table S1: Salmonella outbreak isolates used as reference data set for phylogenetic analysis (Timme et al., 2017).

Strain	GenBank Assembly	SRA Accession	Outbreak year-Location	Location
CFSAN000212	GCA_000748245.1	SRR500494	2005	UAE
CFSAN000211	GCA_000698715.1	SRR498373	2005	Thailand
CFSAN000191	GCA_000698635.1	SRR498369	2005	Thailand
CFSAN000228	GCA_000748565.1	SRR500493	2006	Taiwan
CFSAN000189	GCA_000439415.1	SRR498276	2003	India
CFSAN000963	GCA_000749295.1	SRR498436	2012	USA
CFSAN001118	GCA_000748065.1	SRR1258443	2012	Indonesia
CFSAN000661	GCA_000698515.1	SRR498397	2012	USA
CFSAN000968	GCA_000698535.1	SRR498442	2012	USA
CFSAN001140	GCA_000748085.1	SRR1258440	2012	India
CFSAN001112	GCA_000748025.1	SRR1258439	2012	India
CFSAN000970	GCA_000749415.1	SRR498444	2012	USA
CFSAN000669	GCA_000749005.1	SRR498399	2012	USA
CFSAN000700	GCA_000749045.1	SRR498402	2012	USA
CFSAN000752	GCA_000749065.1	SRR498403	2012	USA
CFSAN000951	GCA_000749145.1	SRR498422	2012	USA
CFSAN000753	GCA_000749085.1	SRR498404	2012	USA
CFSAN001115	GCA_000748045.1	SRR1258442	2012	India
CFSAN000954	GCA_000748405.1	SRR498425	2012	USA
CFSAN000952	GCA_000749165.1	SRR498423	2012	USA
CFSAN000960	GCA_000748505.1	SRR498433	2012	USA
CFSAN000958	GCA_000748465.1	SRR498431	2012	USA
CFSAN000961	GCA_000748525.1	SRR498434	2012	USA

Supplemental Table S2: Resource usage for main steps in the data analysis. The amount of central processing unit hours and the amount of memory used in Gigabytes (GB) for steps of the workflow is provided for assembly without polishing and assembly after two rounds of polishing.

Processor usage (CPU hours)			
**Step**	**No Nanopolish**	**One Round of Nanopolish**	**Two rounds of Nanopolish**
Unicycler	8.8	8.8	8.8
Nanopolish	0	359.6	729.7
Other	4.5	4.5	4.5
**Maximum memory usage (GB)**			
**Step**	**No Nanopolish**	**One round of Nanopolish**	**Two Rounds of Nanopolish**
Unicycler	5.4	5.4	5.4
Nanopolish	0	1	1
Other	5.2	5.2	5.2
